# Co-evolutionary networks of genes and cellular processes across fungal species

**DOI:** 10.1186/gb-2009-10-5-r48

**Published:** 2009-05-05

**Authors:** Tamir Tuller, Martin Kupiec, Eytan Ruppin

**Affiliations:** 1 School of Computer Sciences, Tel Aviv University, Ramat Aviv 69978, Israel; 2 Department of Molecular Microbiology and Biotechnology, Tel Aviv University, Ramat Aviv 69978, Israel; 3 School of Medicine, Tel Aviv University, Ramat Aviv 69978, Israel

## Abstract

**Background:**

The introduction of measures such as evolutionary rate and propensity for gene loss have significantly advanced our knowledge of the evolutionary history and selection forces acting upon individual genes and cellular processes.

**Results:**

We present two new measures, the 'relative evolutionary rate pattern' (rERP), which records the relative evolutionary rates of conserved genes across the different branches of a species' phylogenetic tree, and the 'copy number pattern' (CNP), which quantifies the rate of gene loss of less conserved genes. Together, these measures yield a high-resolution study of the co-evolution of genes in 9 fungal species, spanning 3,540 sets of orthologs. We find that the evolutionary tempo of conserved genes varies in different evolutionary periods. The co-evolution of genes' Gene Ontology categories exhibits a significant correlation with their functional distance in the Gene Ontology hierarchy, but not with their location on chromosomes, showing that cellular functions are a more important driving force in gene co-evolution than their chromosomal proximity. Two fundamental patterns of co-evolution of conserved genes, cooperative and reciprocal, are identified; only genes co-evolving cooperatively functionally back each other up. The co-evolution of conserved and less conserved genes exhibits both commonalities and differences; DNA metabolism is positively correlated with nuclear traffic, transcription processes and vacuolar biology in both analyses.

**Conclusions:**

Overall, this study charts the first global network view of gene co-evolution in fungi. The future application of the approach presented here to other phylogenetic trees holds much promise in characterizing the forces that shape cellular co-evolution.

## Abstract 2

Two new measures of evolution are used to study co-evolutionary networks of fungal genes and cellular processes; links between co-evolution and co-functionality are revealed.

## Background

The molecular clock hypothesis states that throughout evolutionary history mutations occur at an approximately uniform rate [1,2]. In many cases this hypothesis provides a good approximation of the actual mutation rate [2,3] while in other cases it has proven unrealistic [2,4]. The evolutionary rate (ER) of a gene, the ratio between the number of its non-synonymous to synonymous mutations, *dN*/*dS*, is a basic measure of evolution at the molecular level. This measure is affected by many systemic factors, including gene dispensability, expression level, number of protein interactions, and recombination rate [5-11]. Since the factors that influence evolutionary rate are numerous and change in a dynamic fashion, it is likely that the evolutionary rate of an individual gene may vary between different evolutionary periods. Previous studies have investigated co-evolutionary relationships between genes on a small scale, mainly with the aim of inferring functional linkage [12-17]. These studies were mostly based on the genes' phyletic patterns (the occurrence pattern of a gene in a set of current organisms). Recently, Lopez-Bigas *et al. *[18] performed a comprehensive analysis of the evolution of different functional categories in humans. They showed that certain functional categories exhibit dynamic patterns of sequence divergence across their evolutionary history. Other studies have examined the correlations between genes' evolutionary rates to predict physical protein-protein interactions [19-24]. A recent publication by Juan *et al. *[24] focused on *Escherichia coli *and generated a co-evolutionary network containing the raw tree similarities for all pairs of proteins in order to improve the prediction accuracy of protein-protein interactions. Here our goal and methodology are different; we concentrate on a set of nine fungal species spanning approximately 1,000 million years [25]. We develop tools to investigate co-evolution in both conserved and less-conserved genes. For the first group, whose members have an identical phylogenetic tree, we employ high-resolution ER measures to investigate gene co-evolution. In the case of less conserved genes, we generalize the concept of propensity for gene loss [17] to encompass the whole phylogenetic tree in order to better understand the driving forces behind co-evolution.

The first part of this paper describes the analysis of conserved genes. We define a new measure of co-evolution for such genes and study their evolutionary rates along different parts of the evolutionary tree. Next, we reconstruct a co-evolutionary network of genes and a co-evolutionary network of cellular processes according to this measure. In such a network two genes/processes are connected if their co-evolution is correlated. We identify two patterns of co-evolution, correlated (cooperative) and anti-correlated (reciprocal). We show that co-evolution is significantly correlated with co-functionality but not with chromosomal co-organization of genes. We conclude this part by identifying clusters of functions in the co-evolutionary network. Subsequently, in the second part of the paper, we study the evolution of less-conserved genes. We describe a new measure of evolution for such genes and reconstruct a co-evolutionary network of cellular processes according to this measure. We study the resulting clusters in this network and compare it to the co-evolutionary network of the conserved genes.

## Results and discussion

### The co-evolution of conserved genes

#### Computing the relative evolutionary rate pattern

**Figure 1:**
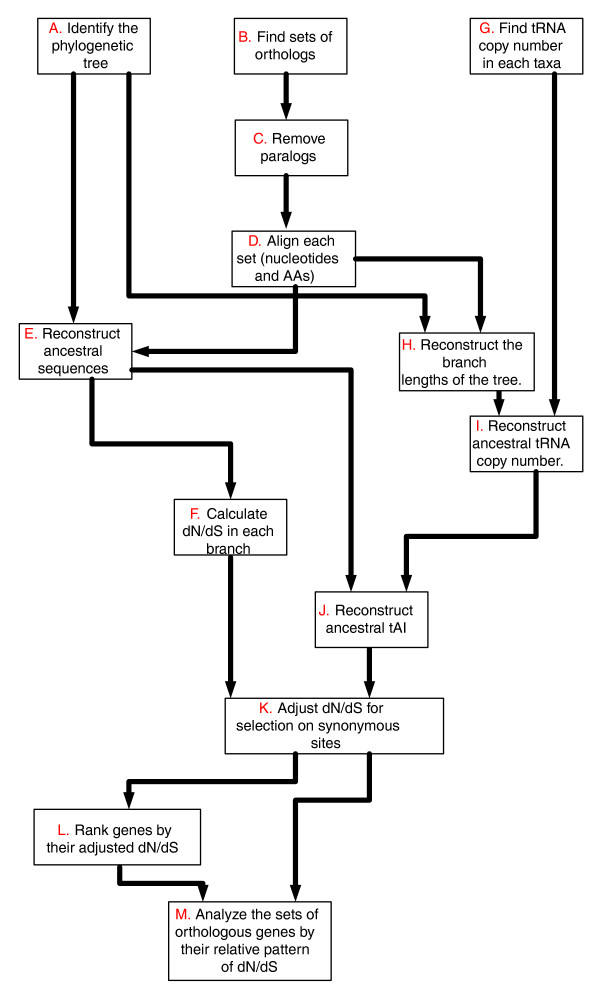
The different steps in computing rERP (for additional details see the Materials and methods section). AA, amino acids; tAI, tRNA adaptation index.

**Figure 2:**
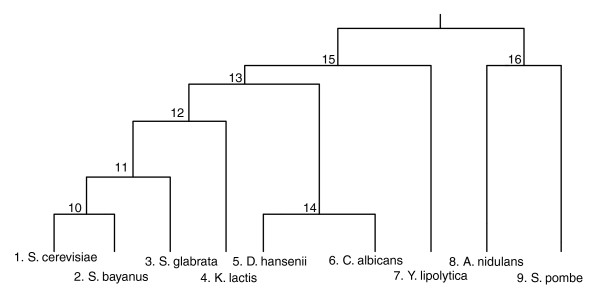
Phylogeny of the 9 fungal species based on the 18S rRNA [26] and 531 concatenated proteins [27]. Each of the leaves and the internal nodes is labeled with numbers between 1 and 15. A branch in the phylogenetic tree is designated by the two nodes it connects.

First, we focus on the large set of conserved genes (that is, genes that are conserved in all fungal species analyzed), identifying sequence co-evolutionary relationships that are manifested in the absence of major gene gain and loss events. As these co-evolutionary relationships cannot be deciphered by an analysis based on phyletic patterns, and a single evolutionary rate measure is too crude for capturing them, we set out to measure the relative evolutionary rate of each gene at every branch of the evolutionary tree. The resulting new 'relative evolutionary rate pattern' (rERP) measure characterizes a gene's pattern of evolution as a vector of all its relative evolutionary rates in the different branches of a species' phylogenetic tree. A workflow describing the determination of genes' ERPs is presented in Figure 1 (for a detailed description of the workflow described in this figure and comparison to other measures of co-evolution see Materials and methods). We analyzed genes from nine fungal species, whose phylogenetic relationship (based on the 18S rDNA [26] and on the comparison of 531 informative proteins [27]) is presented in Figure 2. We first created a set of orthologous genes (lacking paralogs) that are conserved in all species, resulting in a dataset of 1,372 sets of orthologs spanning a total of 12,348 genes. Each such set of orthologous genes (SOG) was then aligned, and its ancestral sequences at the internal nodes of the phylogenetic tree were inferred using maximum likelihood. The resulting sets of orthologs and ancestral sequences were then used to estimate the evolutionary rate, *dN*/*dS *[28], along each of the tree branches. To consider the selection forces acting on synonymous (*S*) sites we used an approach similar to that of [29] and adjusted the evolutionary rates accordingly. These adjusted evolutionary rates are denoted *dN*/*dS'*, and compose an ERP vector that specifies a *dN*/*dS' *value for each branch of the evolutionary tree, for each SOG. We next carried out an analysis of the resulting ERP matrix, whose rows are the SOGs, its columns are the tree branches, and its entries denote evolutionary rate values (*dN*/*dS'*).

#### The evolutionary rate along different branches of the evolutionary tree

**Figure 3:**
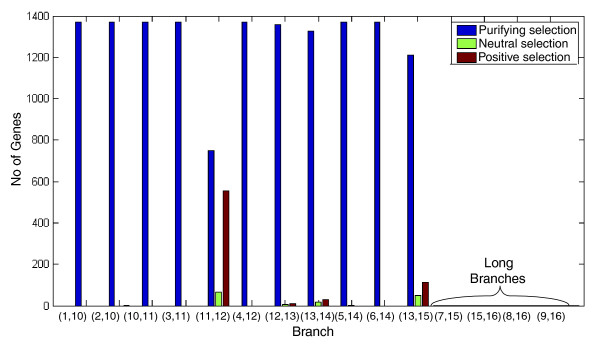
Number of genes (y-axis) with *dN*/*dS' *> 1.1 (positive selection), 1.1 > *dN*/*dS' *> 0.9 (neutral selection), and 0.9 > *dN*/*dS' *(purifying selection) in each branch (x-axis; see Figure 3).

Our first task was to characterize the global selection regimes acting upon the genes studied. We conservatively limit this investigation to the short branches of the tree (excluding branches (7,15), (15,16), (8,16), (9,16); Figures 2 and 3) to avoid potential saturation problems that may bias the ER computation (Materials and methods). Most of the genes exhibit purifying selection (*dN*/*dS' *< 0.9) in the majority of the phylogenetic branches, as one would expect [30]. A much smaller group of genes under positive (*dN*/*dS' *> 1.1) and neutral (0.9 <*dN*/*dS' *< 1.1) selection are concentrated in three branches (Figure 3), with the majority located on the branch leading from internal node 12 to internal node 11, probably following the whole genome duplication event known to have occurred at this bifurcation [31]. This major duplication event probably served as a driving force underlying this surge of positive selection, by relaxing the functional constraints acting on each of the gene copies [32]. This branch also represents a switch from anaerobic (*Saccharomyces cerevisiae*, *Saccharomyces bayanus *and *Candida glabrata*) to aerobic (*Aspergillus nidulans*, *Candida albicans*, *Debaryomyces hansenii*, *Kluyveromyces lactis*, *Yarrowia lipolytica*) metabolism [33], which has likely required a large burst of positive evolution in many genes. Additional data file 1 includes a table that depicts the SOGs with positive evolution along this branch (using their *S. cerevisiae *representative), which is indeed enriched with many metabolic genes. The other two branches under positive selection are the branch between nodes 13 and 14, leading to a subgroup (*D. hansenii *and *C. albicans*) that evolved a modified version of the genetic code [34], and the branch between nodes 13 and 15 that leads to *Y. lipolytica *(which is a sole member in one of the three taxonomical clusters of the Saccharomycotina [35]).

#### Co-evolution of cellular processes

**Figure 4:**
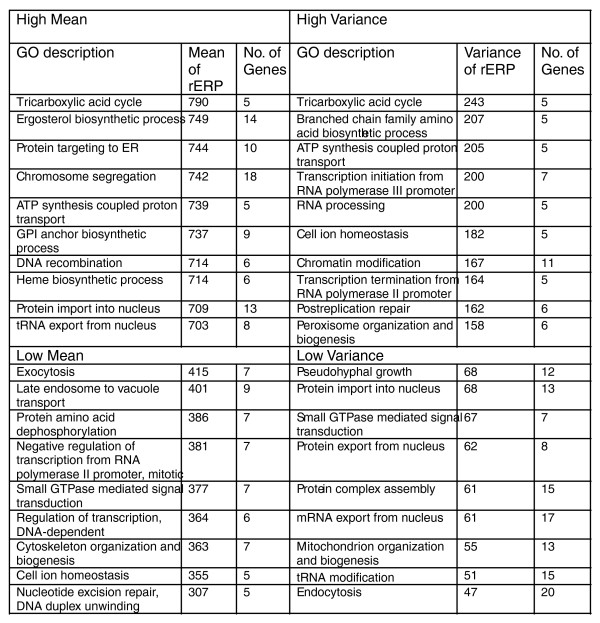
GO categories (biological processes) with extreme mean and variance of their rERPs (for a unbiased comparison we included only GO groups with 5 to 20 genes).

The major goal of this work is to study the co-evolution of gene pairs and of cellular processes. To this end we utilized the ERP matrix to compute the rERP of each conserved SOG. The rERP is a vector containing the relative, ranked *dN*/*dS' *(rER) of each SOG in every branch of the evolutionary tree, thus comparing the evolutionary rate of each individual SOG to that of all other SOGs. The ranking procedure is employed to attenuate the effects of noisy estimations of ER values, especially in long branches of the phylogenetic tree (see Note 1 in Additional data file 2). Defining the rERP of a Gene Ontology (GO) process to be the mean rERP of all the genes it contains, we asked which GO processes have the rERP with the highest mean and the highest variance across the different branches of the evolutionary tree (Figure 4). Notably, processes related to energy production, such as the tricarboxylic acid cycle (involved in cellular respiration), and ATP synthesis-coupled proton transport (which includes genes encoding the mitochondrial ATPase) have the highest mean rERP and also exhibit the highest variance of their rERP. This reflects the primary role that energy production has played in fungal evolution, and the effects that changes from anaerobic to aerobic metabolism have had on the development of fungal species. Additional high rERP energy-related GO terms include aerobic respiration and heme biosynthesis. Interestingly, biological functions related to information flow within the cell exhibit high mean rERP values (tRNA export from nucleus, DNA recombination) or high rERP variance (transcription initiation from polymerase II promoter, RNA processing, transcription termination from RNA polymerase II promoter). The trend, however, is not identical for all processes: protein import to the nucleus, for example, has a high rERP value but very little variance. Full lists of conserved genes and GO groups sorted according to their mean rERP and rERP variance appear in Additional data file 3.

**Figure 5:**
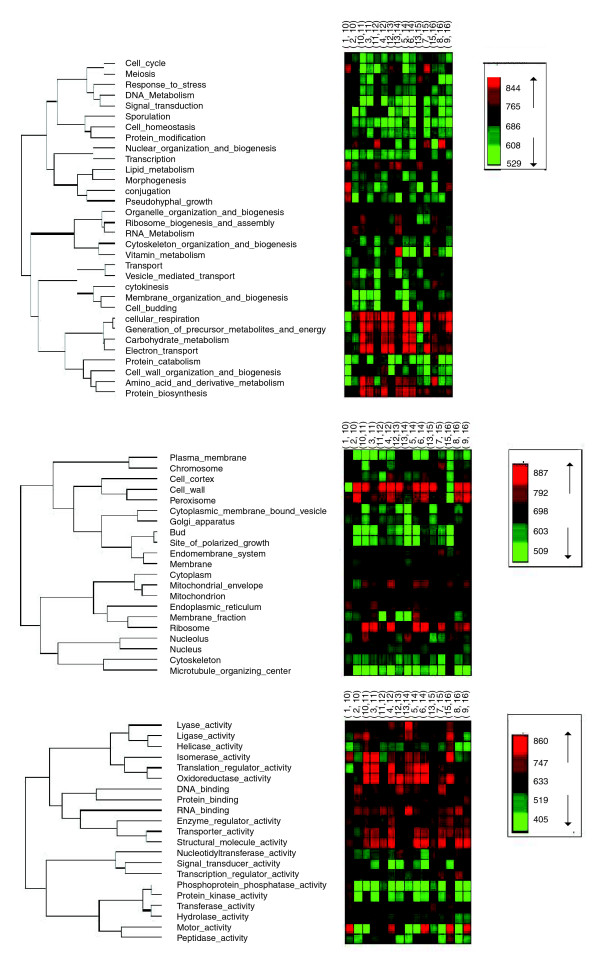
Hierarchical clustering of GO groups (for biological process (top), cellular component (middle), and molecular function (bottom)) according to their rERPs.

**Figure 6:**
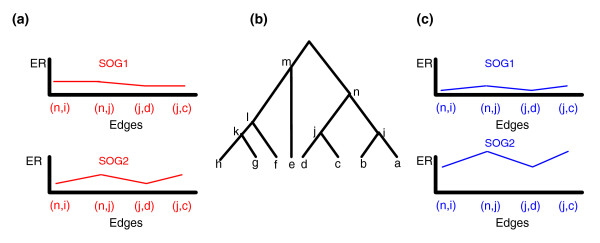
Two hypothetical examples that demonstrate the difference between measuring co-evolution using rERP and applying the average ER along the entire evolutionary tree. **(a) **An example in which ER is high but rERP is low: two SOGs (in red) have similar average ER (|E1 - E2| is small) but the correlation between their ERP vectors is low. Note that the level of co-evolution is low in both cases, but the pattern along the phylogenetic tree is very different. **(b) **A hypothetical evolutionary tree. **(c) **An example in which ER is low but rERP is high: two SOGs (in blue) have similar ERPs but their mean ERs are different. In this case a similar pattern can be seen despite very different levels of ER.

**Figure 7:**
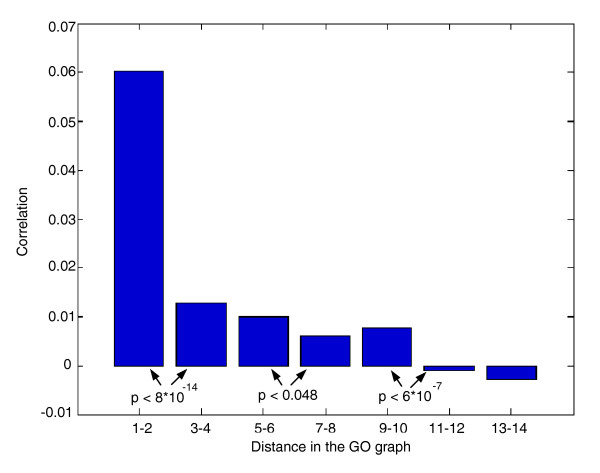
Average correlation between the evolutionary patterns of pairs of GO groups (y-axis) as a function of their distance (the shortest connecting pathway) in the GO network (x-axis). The distribution of correlations in three out of six consecutive pairs of distance bins is significantly different (*t*-test, *P *< 0.05). The correlation between distance (x-axis) and average correlation (y-axis) is -0.96 (*P *< 4.5 × 10^-4^; a similar result was observed when we used the ontology of *S. pombe *(Additional data file 4)). The increase distance 9-10 though deviating from the overall trend is not significant (*P *= 0.23).

We carried out a hierarchical clustering of GO-slim functions according to their rERP values, which is depicted in Figure 5. Many GO-slim groups exhibit correlated rERP values. For example, processes related to metabolic activity (such as cellular respiration, carbohydrate metabolism, and generation of precursor metabolites and energy) exhibit high rERP values across the tree, whereas others (cell cycle and meiosis) exhibit markedly lower values. Interestingly, processes related to polarized growth and budding exhibit the lowest overall rERPs. Importantly, the figure shows that rERP values can provide additional information to that contained in the global relative evolutionary rates (that is, those measured by aggregating the whole tree). For example, the two GO-slim process groups plasma membrane and microtubule organization center (Figure 5, middle) have relatively similar (low) relative global evolutionary rates but markedly different rERPs (as they appear in the two extreme parts of the hierarchical clustering). While the standard ER measure checks if the average ER of genes is similar (that is, |ER_1 _- ER_2_|), rERP compares the fluctuations in the ER of genes. Thus, two SOGs may appear similar by one measure and very different when applying the other. Figure 6 shows two examples in which the two measures provide opposite results. Notably, the correlation between these two measures is significant but rather low (r = -0.055, *P *< 10^-16^). Overall, GO groups with functionally related gene sets (that is, those that map closer on the GO ontology network) tend to have similar rERP values (the correlation between distance in the GO graph and average correlation of rERP is -0.96, *P*-value < 4.5 × 10^-4^; see more details in Figure 7, Additional data file 4, and Materials and methods; this comparison is made using the *S. cerevisiae *GO ontology and mapping all the SOGs to this ontology).

#### Two fundamental types of co-evolution

**Figure 8:**
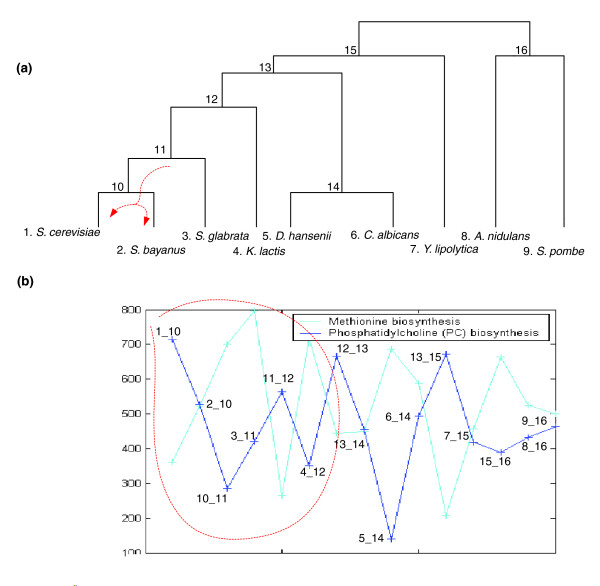
An illustrative example involves the category of methionine biosynthesis, which has a negative rERP correlation with phosphatidylcholine (PC) biosynthesis, an important and abundant structural component of the membranes of eukaryotic cells. PC is synthesized by three successive transfers of methyl groups from S-adenosyl-methionine to phosphatidyl-ethanolamine [36,37]; thus, the evolution of PC biosynthetic pathways may be conditioned by the evolution of methionine biosynthesis pathways, and follow it by some time lag. This phenomenon is demonstrated in the subtree below internal node 11 **(a)**. The rERPs of these two GO functions are shown in **(b)**.

Having a representative rERP vector for each SOG/process enables us to examine the correlations between them and to learn about their co-evolutionary history. A positive rERP correlation arises when two SOGs/processes exhibit a similar pattern of change in the different branches of the evolutionary tree and have evolved in a coordinated, cooperative C-type fashion. A simple example of such a co-evolution is the mitochondrial genome maintenance and mitochondrial electron transport categories. A marked negative rERP correlation denotes reciprocal, R-type co-evolution where periods of rapid evolution of one SOG/process are coupled with slow evolution in the other; this may arise when the rapid evolution of one process creates a new niche or biochemical activity that, in turn, enables, or selects for, the rapid evolution of the other process. An illustrative R-type example involves the category of methionine biosynthesis, which has a negative rERP correlation with phosphatidylcholine (PC) biosynthesis. PC is synthesized by three successive transfers of methyl groups from S-adenosyl-methionine to phosphatidyl-ethanolamine [36,37]. Thus, the evolution of the PC biosynthetic pathway may be conditioned on the evolution of the methionine biosynthesis pathway, and thus follow it with some time lag (Figure 8). Interestingly, genes that co-evolve in a C-type manner do provide functional backups to each other, having a statistically significant enrichment in genetic interactions (hypergeometric *P*-value < 0.0039), while genes co-evolving in an R-type manner do not (where the enrichment is studied using the *S. cerevisiae *genes in each of the pertaining SOGs). We also found that the fraction of sequence-similar SOGs is significantly larger among pairs of C-type co-evolving genes than among pairs of R-type co-evolving genes (Note 2 in Additional data file 2).

#### Co-evolutionary network of SOGs and its properties

**Figure 9:**
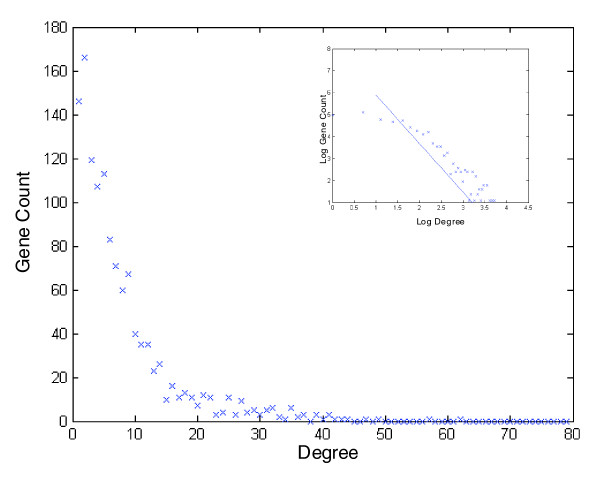
The degree distribution in the co-evolution network is not far from a power-law (the plot of the log(number of genes) as a function of the log(degree) appears in the right-upper corner. The correlation between these two measures is -0.77, *P *= 7.4 × 10^-11^.

To track down the co-evolution of SOGs, we generated a co-evolution network where two SOGs (termed, for convenience, according to the *S. cerevisiae *genes they contain) are connected by an edge only if there is a significant (either positive or negative) Spearman rank correlation (with *P *< 0.05) between their rERPs. The node degrees in the co-evolution network follow a power-law distribution (Figure 9) and the network has small world properties (the average distance between two nodes is 5.03). Many biological networks (for example, see [38,39]) exhibit similar properties. The degree in the co-evolutionary network is significantly correlated with the degree in the *S. cerevisiae *protein interaction network (r = 0.0726, *P *= 0.0125) but is not significantly correlated with the degree in the *S. cerevisiae *genetic interaction network, or with the degree in its gene expression network.

#### Co-evolution is correlated with similar functionality

A co-evolution network of cellular functional categories was built for each of the three GO ontologies (biological process, cellular component, molecular function), using two significance cutoff values (Spearman *P*-value < 0.01 and Spearman *P*-value < 0.001) to determine significant correlations between GO categories. A list of highly correlated pairs of GO terms is provided in Additional data file 5. The correlation between the distance of GO groups in the 0.001 cutoff co-evolution network (that is, their evolutionary distance) and their distance in the corresponding GO ontology network (that is, their functional distance) is highly significant: 0.38 for cellular component, 0.16 for biological process and 0.43 for molecular function (all three with *P*-values <10^-16^; a similar trend is observed using the 0.01 cutoff network). A similarly marked correlation between evolutionary and functional relationships of GO groups is also found when considering positive and negative co-evolution networks separately (Note 3 in Additional data file 2).

Similar results were observed when we considered classification according to Enzyme Commission (EC) number [40], which is a numerical classification scheme for enzymes based on the chemical reactions they catalyze. By this classification, the code of each enzyme consists of the letters 'EC' followed by four numbers separated by periods. Those numbers represent progressively finer classifications of the enzyme. Thus, it induces a functional distance. Our analysis shows that pairs of orthologs with smaller functional distance (genes whose first two roughest classification levels are identical) exhibit higher levels of correlation between their rERP than other pairs of orthologs (mean rERP correlation of 0.31 versus 0.27, *P *= 1.23 × 10^-7^).

#### Co-evolutionary score and other properties of cellular functions and SOGs

We did not find a parallel significant correlation between the genomic co-localization of GO groups and their co-evolutionary score (see Materials and methods for a description of how we computed the co-localization score of pairs of GO groups). The co-evolution of genes and their chromosomal location are not correlated even when considering each chromosome separately. Thus, we conclude that cellular functionality is a more important force driving gene co-evolution than their genomic organization.

The rERP measure correlates well with other systemic qualities such as genetic and physical interactions. The average Spearman correlation between rERP levels of interacting proteins in the *S. cerevisiae *protein interaction network is 0.063, which is 155 times higher than the average correlation (4.05 × 10^-4^) for non-interacting proteins (*P *< 10^-16^). Proteins that are part of a complex show a correlation of 0.05 between their rERPs, 100 times higher than the average correlation for proteins that are not a part of the same complex (*P *< 10^-16^). The Spearman correlation between rERP levels of genetically interacting proteins is 0.02, which is 32 times higher than the average correlation (6.08 × 10^-4^) for non-interacting proteins (*P *= 2.71 × 10^-6^). Protein rERPs are also correlated with the co-expression of their genes (Spearman correlation 0.063, *P *< 10^-16^). The significant correlation between co-evolution and physical/functional interactions suggests that physical interactions between the products of conserved genes play a part in their co-evolution. Namely, to maintain the functionality of an interaction, a change in one protein is likely to facilitate the evolution of the proteins interacting with it, as has already been shown [5]. Yet, as the magnitude of this correlation is rather low, it is likely that other co-evolutionary forces play a part in determining co-evolution, such as the sharing of common and varying growth environments during evolutionary history.

#### Clustering of co-evolutionary networks

**Figure 10:**
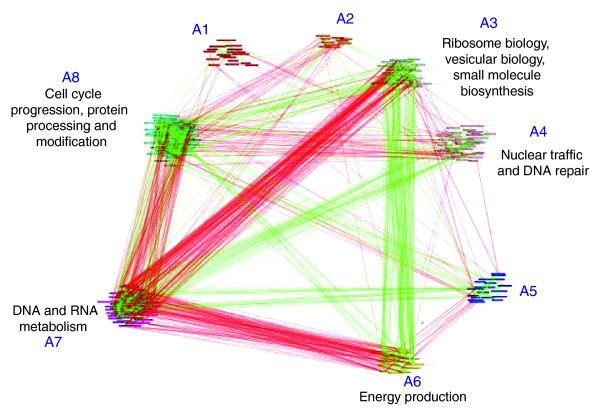
Clustering of biological process GO terms according to their rERP correlations using the PRISM algorithm (with the less stringent significance criterion of *P *< 0.01).

We employed the PRISM algorithm [41] to partition each of the three GO co-evolution networks (biological process, cellular component, molecular function) into clusters of nodes, such that nodes from one cluster have similar sign connections (denoting positive or negative rERP correlations) with nodes from other clusters. We focus here on biological processes at a significance cutoff value of *P *< 0.01 (Figure 10). PRISM clusters the process terms into coherent groups in a statistically significant manner (*P *< 0.001; see Materials and methods), where most of the groups are enriched for particular types of processes: Cluster A7 contains many processes related to DNA metabolism, chromatin formation and RNA processing. This cluster shows strong negative correlations with clusters A6 (amino acid biosynthesis, tricarboxylic acid cycle, glucose oxidization and energy production) and cluster A8 (protein processing and modification). It has also strong positive correlations with cluster A4 (nuclear traffic and DNA repair) and with cluster A5. We note that among the RNA-related processes in cluster A7, some (such as mRNA export from nucleus and poly-A dependent mRNA degradation) show R-type correlations with functions such as protein degradation via the multivesicular pathway. This relationship points to a mode of evolution in which the two catabolic processes (protein and RNA) require coordination, so that changes in one are dependent on preceding changes in the other. Similarly, cluster A6 shows strong coordinated co-evolution with cluster A3 (amino acid and purine biosynthesis, glucose oxidization, energy production and ribosome biology). Both clusters include GO functions related to the production of energy and, thus, coordinated evolution is expected. An overview of the results shows that genes that affect regulatory or information-related processes (DNA metabolism, chromatin formation and RNA processing (cluster A7)) are 'master players'. These master genes/processes exert reciprocal selection forces on many other metabolic process (clusters A8, A3 and A6) and participate in the co-evolution of other processes such as nuclear traffic (cluster A4).

### Co-evolution of less conserved genes

#### The copy number pattern measure

The results presented above were focused on the analysis of a conserved set of genes whose orthologs appear in all nine fungal species studied, comprising 1,372 SOGs and spanning a total of 12,348 genes. The fungal dataset additionally includes 2,168 orthologous sets spanning more than 74,851 genes that exhibit at least one change in their copy number along the phylogenetic tree (and hence have undergone gene loss and/or gene duplication events). The 'propensity for gene loss' (PGL) [17] was shown to correlate with gene essentiality, the number of protein-protein interactions and the expression levels of genes. PGL has been used in methods for predicting functional gene linkage [42,43], extending upon previous methods that used the occurrence pattern of a gene in different organisms for the same aim [12-14]. Recently, a probabilistic approach related to the PGL was developed [42]. A related measure, which is also based on a gene's phyletic pattern (the occurrence pattern of a gene in different current organisms), is phylogenetic profiling (PP) [15,16,43]. This measure has been employed in previous small scale studies to identify sets of genes with a shared evolutionary history [12-15,43]. We describe a new measure of co-evolution that is a generalization/unification of both PGL and PP, termed the copy number pattern (CNP). Like PP, it characterizes each gene by examining its phyletic pattern (but additionally takes into account the number of paralogous copies of each gene in the genome). Like PGL, it exploits the information embedded in a species' phylogenetic tree to more accurately characterize the evolutionary history of each gene (in comparison, PP carries out a similar computation based on just the phyletic pattern). We used the new CNP measure to analyze orthologous sets that exhibit at least one change in copy number along the analyzed phylogentic tree. This set of genes is, by definition, not completely conserved, and complements the conserved set of genes analyzed by the rERP measure.

**Figure 11:**
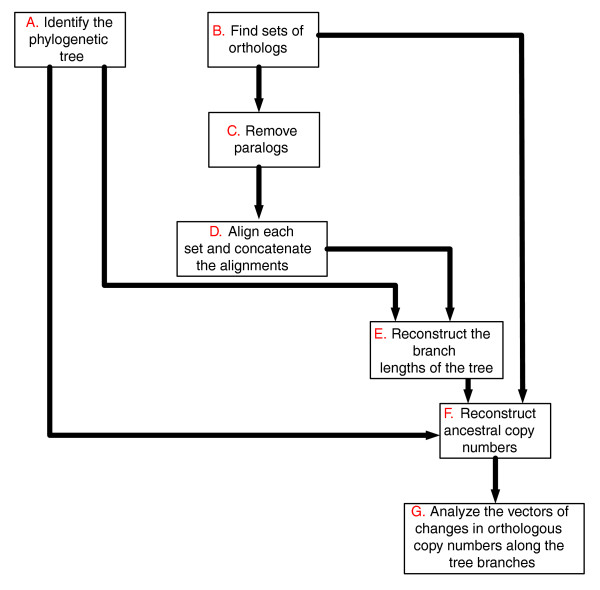
The different steps in computing CNP (a detailed description is provided in the 'Co-evolution of less conserved genes' section).

Figure 11 provides a stepwise overview of CNP computation. Steps A to F are essentially similar to those used to generate the rERP (see Materials and methods): we first generate a set of 2,168 orthologous sets that exhibit at least one change in copy number along the analyzed phylogentic tree. We then translate the resulting set of orthologs to copy numbers in each organism (the number of paralogs in an organism), and reconstruct the ancestral gene copy number using CAFE' [44] (step F in Figure 11). Finally, using the copy number in each internal node, we compute the change (the difference) in copy number for orthologous sets along each edge (step G in Figure 11). The orthologous sets used for the rERP analysis and the orthologous sets used for the CNP analysis include two different, exclusive groups of conserved versus less-conserved genes, respectively. Analysis of these sets reveals that the first group is enriched with metabolic processes while the second is enriched with functional processes such as reproduction and cell differentiation (Additional data file 6).

#### Co-evolution of less conserved genes with the copy number pattern measure

Since changes in the copy number of genes are infrequent events, the Spearman correlations between pairs of CNP vectors are usually very high (the average Spearman correlation is 0.63). To overcome this, we generated CNP vectors of GO processes (according to the biological processes ontology) where the CNP of a GO category is the mean CNP of all the genes it contains. These GO process vectors exhibit a wider range of CNP values. Next, we constructed a GO process co-evolution network. In this network two biological processes are connected by an edge only if they manifest an extreme co-evolution pattern - that is, if they have a Spearman rank correlation that is higher (green colored edges, denoting coordinated relationships) or lower (red, denoting reciprocal relationships) than the correlation values of X% of the total GO pairs. We examined the networks formed under two edge-selection regimes, a more stringent one where X% = 99.9% and a less stringent one where X% = 98%. The correlation between the distance of GO groups in the network with X% = 99.9% and the distance of GO groups in the different GO ontology networks is highly significant (r = 0.4209, *P *< 10^-16^) for green, cooperative edges, and negatively correlated (r = -0.12, *P *< 0.04) for red, reciprocal edges. This suggests that the two types of edges are informative: the green edges represent functional relationships while the red ones represent pairs of GOs with distant functions.

#### Clustering of the copy number pattern evolutionary network

**Figure 12:**
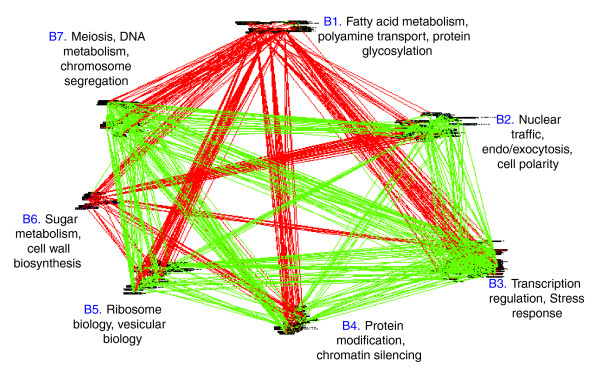
Clustering of biological process GO terms according to their CNP using the PRISM algorithm (*P *< 0.04).

To learn more about the structure of the CNP co-evolution network, we used the PRISM algorithm [41] (as in the case of the rERP analysis) to partition each of the GO terms in the less stringent network (X% = 98%; thus obtaining a larger amount of edges and a more robust clustering) into clusters of nodes, such that nodes from one cluster have similar color edge connections with nodes from other clusters. PRISM is able to separate the process terms into coherent groups according to their mutual correlations, in a statistically significant manner (*P *< 0.001; see Materials and methods). Figure 12 displays the results of the PRISM analysis, clustering of the GO biological processes into seven large groups. The seven interconnected groups are enriched with specific processes, and present clear interactions of the red and green edges. As clearly seen in Figure 12, some clusters show mainly reciprocal co-evolution with most of the others (for example, clusters B1 (fatty acid metabolism) and B6 (sugar metabolism). In contrast, clusters B2, B3 and B7 (nuclear traffic, transcription and DNA metabolism) show coordinated (C-type) co-evolution. Cluster B4 (protein modification, chromatin silencing) shows C-type co-evolution with transcription (cluster B3) and nuclear import (cluster B2) as well as DNA metabolism (cluster B7) but R-type relations with cluster B1, which includes fatty acid metabolism and protein glycosylation.

#### Comparison of the co-evolution of conserved versus less-conserved genes

A comparison between the results obtained by the rERP and CNP methodologies at a global level should be done with some caution, for three main reasons. First, these two measures are applicable for the analysis of completely disjoint, complementary sets of orthologs. Second, the two methodologies measure different types of co-evolution. The rERP measures evolution via amino acid substitutions while the CNP measures co-evolution via changes in gene copy number, which are mainly driven by gene gain and loss events. Thus, third, these co-evolutionary relationships are possibly the result of the action of different evolutionary forces. However, it may be noted that some biological processes present the same type of evolutionary relationship with both methods. For example, DNA metabolism is always positively correlated with nuclear traffic, transcription and vacuolar biology (ER-Golgi traffic). Yet, some of the clusters exhibit different relationships when analyzed by the two measures. For example, a cluster containing mainly genes labeled ribosome biology and vacuolar biology exhibits reciprocal evolution with DNA metabolism by rERP (clusters A3 to A7) but coordinated evolution by CNP (clusters B5 to B7). Thus, within a certain biological process, the evolutionary pressures exerted on highly conserved genes may differ from those that apply to less conserved ones, and may thus provide different opportunities for co-evolution.

## Conclusions

Our analysis charts the first global network view of the co-evolution of conserved and less conserved genes in nine fungal species. We find that cellular functions play a more important driving force in gene co-evolution than the genes' chromosomal location. Two fundamental patterns of co-evolution, cooperative and reciprocal, are defined, and, remarkably, we find that only genes co-evolving cooperatively functionally back each other up. At the single gene level, the observation that genes have evolved at accelerated rates in a localized manner on only three branches of the fungal tree is in line with previous findings suggesting that a large fraction of DNA mutations can be attributed to punctuated evolution [4]. The fungal tree analyzed here is a natural starting point. The future application of the approach presented here to other phylogenetic trees, including the mammalian one, holds much promise in characterizing the forces that shape cellular co-evolution.

## Materials and methods

### Data sources

The GO functional classification used in this work is the most comprehensive, qualitative, and widely used annotation database [45].

The GO and GO slim annotations and protein composition data for complexes were downloaded from the *Saccharomyces *Genome Database [46]. We checked and report results both for the GO slim classification (Figure 5), the roughest level of classification, and the general GO ontology (Figures 4, 7, 8, 10, and 12), where we filtered GO groups that were too small (with less than five SOGs in our dataset). That is, the main bulk of the analysis was performed across the whole GO ontology, without focusing on any arbitrary level. Note 4 in Additional data file 2 and Additional data file 7 includes statistics and error rates of the annotations used in this work.

The genetic interaction network data were downloaded from the BioGrid database [47]; similar results were obtained when the genetic interaction network of Tong *et al. *[38] was used (data not shown). Recent work showed that only 5% of the genetic interactions are conserved in *S. cerevisiae *and *Caenorhabditis elegans *[48]. Note that the evolutionary distances between these species (1,542 million years, according to [17]) are much larger than those between the organisms in our dataset (20 to 837 million years). Further, *C. elegans *is multi-cellular while all the analyzed fungi are unicellular. Thus, it is not clear how the conclusions of Tischler *et al. *[48] are related to our dataset.

More importantly, in this work we study the relationship between the genetic interaction network and its co-evolutionary network for one organism (*S. cerevisiae*) for which we know the genetic interactions network. As there is no reason to believe that this organism is 'special' in any way, we believe that these findings are representative of the expected findings for other organisms if or when their genetic interaction networks become known.

The protein interaction network of the budding yeast *S. cerevisiae *was downloaded from the BioGrid database [47].

Gene expression data were taken from the Stanford MicroArray Database [49]. The GO ontology network of yeast was downloaded from the Open Biomedical Ontologies Foundry ontologies [50]. EC numbers of the analyzed genes were downloaded from the Kyoto Encyclopedia of Genes and Genomes (KEGG) [51].

### Computing relative evolutionary rate patterns for orthologous gene sets

The selection of species used here is not arbitrary; obviously, different selections are perhaps equally plausible but several considerations led us to the current selection, which we outline below. For this study, we used fungi whose genomes have been completely assembled (at the time this study was performed: July 2007) according to the National Center for Biotechnology Information (NCBI) and for which we could infer the tRNA gene repertoire reliably and, thus, compute the tRNA adaptation index (tAI). These include *S. cerevisiae*, *C. glabrata*, *K. lactis*, *D. hansenii*, *Y. lipolytica *and *Schizosaccharomyces pombe*. This selection was then augmented by three additional species: *C. albicans*, an important fungal pathogen for which a high-quality gene collection (including tRNA genes) has recently become available [52]; *S. bayanus*, a *Saccharomyces sensu stricto *species that diverged from *S. cerevisiae *approximately 20 million years ago and for which an overwhelming majority of the open reading frames are available [53]; and *A. nidulans*, a filamentous fungus with a high-quality sequence. Furthermore, these species were analyzed recently by Man and Pilpel [33], serving as an appropriate reference set for studying evolutionary events in fungi.

Finally, due to the large evolutionary distance between *S. pombe *and the hemiascomycotic species (350 to 1,000 million years ago [25]), this set of species present a nice distribution of evolutionary time. We believe that small changes in the set of fungi species would likely yield quite similar results (see details in Note 5 in Additional data file 2).

The final dataset included genomes of nine fungal species: *A. nidulans*, *C. albicans*, *C. glabrata*, *D. hansenii*, *K. lactis*, *S. bayanus*, *S. cerevisiae*, *S. pombe*, *Y. lipolytica*.

Computation of the rERPs is a multi-step process (Figure 1 provides an overview), described in detail as follows. The phylogenetic tree used to analyze the data (Figure 2) was formed according to the analysis of 18S rRNA data in [26], the analysis of 531 concatenated proteins [27], and the analysis of additional gene sets listed in [54] (step A in Figure 1). The orthologous sets for the nine fungi were downloaded from [33] (step B in Figure 1). This dataset was generated by the MultiParanoid program [55]. We considered only sets that include orthologs in all nine species. Sets of homologs that did not include exactly one representative in each organism were removed from our dataset to filter out paralogs and avoid potential errors in evolutionary rate estimation due to duplication events (step C in Figure 1). Horizontal gene transfer events (see, for example, [56]) are rare in fungi [35] and thus were not considered in our analysis. The final dataset included 1,372 orthologous sets. Stop codons were removed and each gene was translated to a sequence of amino acids. Each orthologous set was then aligned by CLUSTALW 1.83 [57] with default parameters. By using amino acids as templates for the nucleotide sequences and by ignoring gaps we generated gap-free multiple alignments of the nine orthologous proteins in each orthologous set and their corresponding coding sequences (step D in Figure 1).

Given the alignments of each set of orthologs and given the phylogenetic tree, we used the codeml program in PAML for the joint reconstruction of ancestral codons [58] in each of the internal nodes of the phylogenetic tree (step E in Figure 1). This reconstruction induced the sequence of ancestral proteins and their corresponding ancestral DNA coding sequences. We hence obtained sets of 16 sequences; 9 from the previous step (corresponding to the 9 leaves of the phylogenetic tree; Figure 2) plus 7 reconstructed sequences of the internal nodes of the phylogenetic tree (ancestral nodes 10-16 in Figure 2). We denote such a set of 16 sequences a 'complete ortholgous set'. For each complete ortholgous set, we computed the *dN *and *dS *in each branch of the evolutionary tree using the y00 program in PAML [28,59] (step F in Figure 1). The outputs of this stage are two vectors of 15 positive real numbers for each complete ortholgous set (1,372 pairs of vectors in our case). These vectors denote the *dN *and *dS *values at the 15 different branches of the evolutionary tree.

To adjust for selection on synonymous sites [60], we used a procedure similar to that described in [29], utilizing the tAI [61] instead of the codon adaptation index [33,62]. Following Hirsh *et al. *[29], we assume the following model of evolution on synonymous sites:



where *r*_*0 *_is the neutral evolutionary rate, *k *is a constant, and *t *is time. Our goal is to estimate *dS' = r*_*0 *_× *t*, which is done using regression. This requires the computation of the tAIs of each of the gene sequences (the leaves of the phylogenetic tree), and the estimation of the tAIs of the sequences at the internal nodes of the phylogenetic tree. To this end (step G in Figure 1) we used the tRNA copy number of each species as reported in [33], and the ancestral tRNA copy numbers were reconstructed following [44] (step I in Figure 1) using the CAFÉ program.

The edge lengths (step H in Figure 1) for CAFÉ were computed by the following steps. Step one: we inferred edge lengths under the molecular clock assumption for the tree topology of Figure 2 and the concatenation of all the sets of ortholog proteins (561,072 sites) using the codeml program in PAML [59]. Step two: we normalized the log of branch lengths to obtain branch lengths that are integers between 0 and 1,000 that reflect putative time units (this is the requirement of the method of [44]. Step three: we used an expectation-maximization (EM) algorithm to find the optimal value of λ (0.001756) for the model (see [44]).

It is important to note that by optimizing λ we actually optimize the likelihood of the model, and the result is invariant for the choice of the normalization factor of the branch lengths. The relatively similar tRNA copy number distribution of the nine species [33] also induces a quite similar tRNA copy number distribution at the ancestral nodes of the phylogenetic tree. To compute the tAI of each complete orthologous set (step J in Figure 1), we used the Matlab, R, and Pearl scripts from [61] (see [61] for the exact description of how to compute the tAI).

Thus, by using tAI and *dS *we were able to adjust the *dS *values for selection on synonymous sites, resulting in a new value, *dS*'. This was done for each ortholgous set in each of the tree branches (step K in Figure 1). These *dS*' values were used for computing the corresponding values of adjusted evolutionary rates, *dN*/*dS*'. As mentioned, the idea underlying this step [29] is to assume a linear relationship between *dS *and tAI, and its computation proceeds as follows.

Let *i *(0 <*i *< 1,373) denote an index of a complete ortholgous set, and let *j *(0 <*j *< 16) denote a branch in the phylogenetic tree. We perform the following steps. Step one: for each *i *and *j *compute the average tAI for the sequences at the two ends of the branch *j*; let *tAIi*, *j *denote this average. Step two: for each branch *j *use all the *tAI*_*i*, *j *_and all the *dS*_i, j _(where 0 <*i *< 1,373) and, by regression, estimate *a*_*j *_and *b*_*j *_that minimize the least squares error of the model:



Step three: for each *i *and *j *and an estimation of the substitution rate on synonymous sites *dS*_*i*, *j*_, the adjusted selection on the synonymous sites is:



The final output of this procedure is a total of 1,373 vectors, each with 15 *dN*/*dS*' values denoting the ERP values of each complete orthologous set.

Usually, for very high levels of substitution rate (long branches in the evolutionary tree), the error in the estimated *dS *values increases [63]. This well known phenomenon is named saturation. Thus, we perform an additional normalization of the *dN *values by computing the ranked evolutionary rate, rER. The ranked evolutionary rate, rER (step L in Figure 1), is computed separately for each branch of the evolutionary tree. For a given branch, the rank of the *dN*/*dS' *of a complete ortholgous set among the *dN*/*dS' *values of all the complete ortholgous sets is the number of sets that have lower *dN*/*dS' *values in this branch (a number between 1 and the total number of complete ortholgous sets, 1,373). The rERP of a complete ortholgous set is the vector of its ranked evolutionary rate along the 15 branches of the evolutionary tree. Note 6 in Additional data file 2 and Additional data file 8 include a comparison of the *dN*/*dS' *values to previous evolutionary rate results in a previous study by Wall *et al. *[8].

The rERP developed and used in this work is different from the measure used in [23] in many important ways. We ranked the ER and adjusted the computed *dN*/*dS *for selection on synonymous sites by using the tAI measure (an approach that has not been used before). Additionally, we used the non-parametric Spearman correlation instead of the Pearson correlation. A comparison of the results obtained using our measure with those obtained using Fraser *et al*.'s ER measure for studying co-evolution shows that the ratio between the average correlation of physically interacting genes versus non interacting genes is very low when using Fraser *et al*.'s measure (0.06/0.022 = 2.72), showing a very low discriminative power. In comparison, the ratio obtained using our measure is markedly higher (0.063/4.05 × 10^-4 ^= 155), in correspondence with the expectation that interacting proteins would tend to co-evolve much more than non-interacting ones (for example, see [24]).

Measures that were based only on *dN *instead of *dN*/*dS *performed worse than the rERP mentioned above. Using *dN *without ranking is problematic as longer branches have, in general, higher *dN *and, thus, the correlations obtained were very high and quite similar when comparing genes that physically interact and those that do not (r = 0.77 versus r = 0.73).

When we used ranked *dN *instead of ranked *dN*/*dS*, we achieved better results, which were almost as good as the results we got using rERP, in terms of separating pairs of physically interacting from non-interacting proteins via their co-evolution. For example, the ratio between the correlation of protein-interacting to non-interacting proteins was 20 using ranked *dN*, weaker than the ratio of 155 observed using rERP values.

### Constructing and analyzing the co-evolution networks of GO terms

The co-evolution network of GO terms was constructed as follows. First, consider only GO groups that include at least three genes. Second, compute the rERP of each GO group. Third, compute the Spearman correlation between the rERP of all pairs of GO groups. Fourth, connect a pair of GO groups, G_i _and G_j_, if the following two conditions are satisfied: condition one, they have significant correlation (*P *< 0.01 in the case of the network in Figure 10), where significance is computed empirically versus a corresponding random shuffled network; condition two, the two sets do not strongly overlap in their gene content, having a Jaccard coefficient < 0.5 [64].

The distance between GO terms on the GO network was computed by replacing each directed edge in the original graph with an undirected one, and computing the length of the minimal path between the two GO groups.

The co-evolution network was clustered and visualized using the Matlab implementation of the PRISM algorithm [41]. The PRISM algorithm was instrumental to our analysis as it partitions the graph according to the two types of edges (positive (cooperative) or negative (reciprocal) rERP correlations) to get clusters of nodes, such that nodes from one cluster have edges of a similar type with nodes from other clusters. This is of particular interest when studying co-evolution, since it identifies 'monochromatic' relationships between groups of genes/GO functions - that is, groups of genes that relate to each other in either a completely cooperative or a completely reciprocal manner. To the best of our knowledge, no other method/algorithm is available to achieve this goal. Other clustering algorithms do not preserve the 'monochromatic' property and, hence, are not suitable for addressing the question at hand.

The significance of the monochromaticity of the resulting clustering was computed by comparing the number of conflicts (the number of edges between nodes that are in different clusters and have a color different from that of the majority of edges between the two clusters) in the original clustering to its distribution in 1,000 randomly shuffled networks with similar topological properties.

### Analyzing the genomic co-localization of GO terms

We define the distance between two GO groups as the median of all shortest distances between each gene in one GO group to each gene in the other GO group. We did not consider genes that are common to the two GO groups. For estimating to what extent two GO groups tend to be located close to each other in the genome, we computed a *P*-value based on comparing their median distance to that of a background model obtained by randomly locating all the genes of both groups in the genome, and recomposing their median distance for each such assignment (repeating this process 100 times to obtain a distribution of background model medians). The *P*-value is the fraction of times that a random shift yields a lower distance between the two GO groups.

## Abbreviations

CNP: copy number pattern; EC: Enzyme Commission; ER: evolutionary rate; ERP: evolutionary rate pattern; GO: Gene Ontology; PC: phosphatidylcholine; PGL: propensity for gene loss; PP: phylogenetic profiling; rERP: relative evolutionary rate pattern; SOG: set of orthologous genes; tAI: tRNA adaptation index.

## Authors' contributions

TT carried out all of the analysis. All authors participated in the design of the study. All authors were involved in drafting and writing the manuscript. All authors read and approved the final manuscript.

## Additional data files

The following additional data are available with the online version of this paper: a table that includes the orthologous sets that exhibit positive evolution for each of the tree branches (Additional data file 1); supplementary notes 1 to 6 (Additional data file 2); a table with GO processes with less than 20 genes (biological process ontology) sorted by their mean rERP and variance of rERP (Additional data file 3); a figure that includes the mean correlation between the evolutionary patterns of pairs of GO groups (y-axis) as a function of their distance (the shortest connecting pathway) in the GO network (x-axis) when using the ontology of *S. pombe *(Additional data file 4); a table with pairs of GO groups exhibiting a significant correlation between their rERPs (Additional data file 5); a table with GO enrichments (biological process) for the conserved and non-conserved genes (Additional data file 6); a figure that depicts the distribution of the number of annotations per gene for the conserved and non-conserved genes (Additional data file 7); a figure that depicts the ER values computed in our study versus the ER values computed in Wall *et al. *[8] (Additional data file 8).

## Supplementary Material

Additional file 1 Orthologous sets that exhibit positive evolution for each of the tree branches. Click here for file

Additional file 2 Supplementary notes 1 to 6. Click here for file

Additional file 3 GO processes with less than 20 genes (biological process ontology) sorted by their mean rERP and variance of rERP. Click here for file

Additional file 4 Mean correlation between the evolutionary patterns of pairs of GO groups (y-axis) as a function of their distance (the shortest connecting pathway) in the GO network (x-axis) when using the ontology of *S. pombe*. Click here for file

Additional file 5 Pairs of GO groups exhibiting a significant correlation between their rERPs. Click here for file

Additional file 6 GO enrichments (biological process) for the conserved and non-conserved genes. Click here for file

Additional file 7 Distribution of the number of annotations per gene for the conserved and non-conserved genes. Click here for file

Additional file 8 ER values computed in our study versus the ER values computed in Wall *et al. *[8]. Click here for file
