# Supplementary material for: Co-evolutionary networks of genes and cellular processes across fungal species
Source: Genome Biol. 2009 May 5;10(5):R48. doi: 10.1186/gb-2009-10-5-r48 (PMC2718514; doi:10.1186/gb-2009-10-5-r48)
Supplement: Additional file 2 — Supplementary notes 1 to 6. [file gb-2009-10-5-r48-S2.doc]

# Supplementary Notes for the paper: "Co-evolutionary Networks of Genes and Cellular Processes Across Fungal Species "

*Tamir Tuller, Martin Kupiec & Eytan Ruppin*

***Note 1: dS saturation and ranking ER***

This study focuses on the evolution of conserved genes (i.e. genes that appear in all the analyzed yeasts). On one hand, we aim to use a wide variety of fungi species to obtain high-resolution of ERP measurements. On the other hand, due to saturation, the estimation of dS in the long branches of the resulting large-distance phylogeny can be noisy (Methods). Using ranked values (rER) attenuates this problem, by `normalizing' the ER of each orthologous set according to the ERs of the other orthologous sets in the same branch.

In the long branches, as dS estimations reach their saturation levels, the dS levels of all the orthologous set tend to be similar (see e.g. [63]). Thus, the rER essentially transcribes to a normalization of the dN of each orthologous set according to the

dN of the other orthologous sets.

***Note 2: Evolutionary origin and sequence similarity of co-evolving SOGs***

To investigate whether our dataset includes out-paraglogs (i.e. if there are SOGs that are relatively closely related) we used the COG database [65] and checked if there are pairs of SOGs that appear in the same COG group. We found only 60 such sequence-related SOGs; 9 of them exhibited significant co-evolution (p-value = 0.04); the distribution among the two types of co-evolution was similar (with 4 being R-type and 5 C-type). This paucity of sequence similarity is not surprising as we focused on this part of the analysis on the subset of conserved genes that do not have paralogs in all the 9 fungi studied. Thus, most co-evolution relations observed in our analysis cannot be explained by paralogs relations.

We also performed a similar test using BLAST. First, we checked if significantly co-evolving pairs of SOGs tend to have significantly BLAST score. We didn’t found a significant p-value for this question when we check the two types of co-evolution together or separately.

However, the frequency of SOGs pairs with significant BLAST score was significantly higher for C-type co-evolving pairs than for R-type co-evolving pairs (2% *vs.* 1.5%, Ks p-value = 0.028).

***Note 3: Correlation between the distance of GO categories in the co-evolution network and their distance in the GO ontology networks***

The correlation between the distance of GO groups in the 0.001-cutoff network and the distance of GO groups in the different GO ontology networks is highly significant: r = 0.38 (p-value < 10^-16^) for Cellular Components, r = 0.16 (p-value < 10^-16^) for Biological Processes and r = 0.43 (p-value = 2*10^-16^) for Molecular Functions (and a similar trend is observed using the 0.01-cutoff network).

The results are similar even when taking into account the networks that are created when considering only the negative edges (r = 0.5 with p-value < 10^-16^, r = 0.3517 with p-value < 10^-16^, r = 0.4859 with p-value = 1.19*10^-12^ for the cellular components, biological processes, and molecular functions respectively) or when considering only the positive edges (r = 0.4646 with p-value < 10^-16^, r = 0.2751 with p-value < 10^-16^, and r = 0.4822 with p-value =1.25*10^-13^ for the cellular components, biological processes, and molecular functions respectively).

***Note 4: Statistics about the GO annotation***

All the genes analyzed by the rERP and CNP genes have at least 3 GO annotations. The mean number of annotation for a gene is 6.81 for the genes analyzed by the rERP genes and 5.97 for the genes analyzed by the CNP. Suppl. Figure 2 depicts the distribution of number of annotation for each gene.

The error rate of GO annotations is reasonably low. It was estimated to be around 15.5% (between 13% and 18%) for non-ISS evidence code and around 49% for ISS evidence code [66]. In our dataset the number of ISS based annotations is negligible (only 7%). Thus the overall error rate is estimated to be less than 17.8% = 0.07*49% + 0.93*15.5%. Moreover, it is known that the quality of GO annotation for *S. cerevisiae* is higher than for other organisms (see, for example, [67]). Thus, we believe that the actual error rate in our dataset is much lower.

***Note 5: Robustness of the co-evolutionary network to the analyzed organism set***

To check if the results reported in this study are robust to small changes in the analyzed set of organism we performed the following simulation:

1) We sampled 15 sub-trees with 7 organisms from the original species tree.

2) For each sample, we computed the rERP/CNP that is induced by the corresponding sub-tree.

3) We compared the rERP/CNP co-evolutionary networks that were induced for pairs of samples in two manners:

a) The fraction of pairs of orthologous groups that manifest a significant (p < 0.01) Spearman correlation between their rERP/CNP values in the two co-evolutionary networks.

b) The correlations between the two vectors that denote the Spearman correlation between the rERP/CNP value of all pairs of orthologous groups in the two co-evolutionary networks.

In the case of rERP, on average, 69% of the significantly co-evolving pairs of orthologs appeared jointly in pairs of co-evolutionary networks (p < 10^-16^, measure (a) above). The mean correlation between the corresponding pairwise correlation vectors (measure (b) above) was 0.83 (p < 10^-16^). In the case of CNP, on average, 71% of the significantly co-evolving pairs of orthologs were common to pairs of co-evolutionary networks (p < 10^-16^). The mean correlation between the corresponding pair wise correlations was 0.7 (p < 10^-16^).

These results testify to the robustness to small changes of the results reported.

***Note 6: Comparing the dN/dS' values to ER results from a previous study by Wall et al.***

In a previous study by Wall *et al.* [8] the average *dN*/*dS* along an evolutionary tree of four species (*S. paradoxus*, *S. cerevisiae*, *S. mikatae*, and *S. bayanus*) was computed. Two of these organisms (*S. cerevisiae*, and *S. bayanus*) appear in our dataset. They have a common ancestral node (node 10, see Figure 2A). The Spearman correlation between the *dN*/dS values that were computed by Wall *et al.* [8] and the mean *dN*/*dS* along branches 1_10 and 2_10 by our reconstruction is 0.75 (p-value <10^-50^), a highly marked correlation. For comparison, the correlation between the propensity for gene loss and the evolutionary rate [17] is only about 0.3-0.4. Suppl. Figure 3 provides the plots of the evolutionary rates that were computed in this study vs. the evolutionary rates that were computed in the work of Wall *et al.*
