# Supplementary material for: Co-evolutionary networks of genes and cellular processes across fungal species
Source: Genome Biol. 2009 May 5;10(5):R48. doi: 10.1186/gb-2009-10-5-r48 (PMC2718514; doi:10.1186/gb-2009-10-5-r48)
Supplement: Additional file 4 — Mean correlation between the evolutionary patterns of pairs of GO groups (y-axis) as a function of their distance (the shortest connecting pathway) in the GO network (x-axis) when using the ontology of S. pombe. [file gb-2009-10-5-r48-S4.doc]

**Suppl. Fig 1.** Average correlation between the evolutionary patterns of pairs of GO-groups (y-axis) as a function of their distance (the shortest connecting pathway) in the GO network (x-axis) when using the ontology of *S. pombe*. The correlation between distance (x-axis) and average correlation (y-axis) is -0.93 (p-value = 0.0025). The global trend of lower correlation for higher functional distances is almost perfect (the only deviation from this trend is in the highest functional distance).
