# Supplementary material for: Co-evolutionary networks of genes and cellular processes across fungal species
Source: Genome Biol. 2009 May 5;10(5):R48. doi: 10.1186/gb-2009-10-5-r48 (PMC2718514; doi:10.1186/gb-2009-10-5-r48)
Supplement: Additional file 7 — Distribution of the number of annotations per gene for the conserved and non-conserved genes. [file gb-2009-10-5-r48-S7.doc]

**Suppl. Fig. 2:**  **The distribution of the number of annotations per gene for A. the CNP genes, and B. the rERP genes.**
