# Supplementary material for: Co-evolutionary networks of genes and cellular processes across fungal species
Source: Genome Biol. 2009 May 5;10(5):R48. doi: 10.1186/gb-2009-10-5-r48 (PMC2718514; doi:10.1186/gb-2009-10-5-r48)
Supplement: Additional file 8 — ER values computed in our study versus the ER values computed in Wall et al. [8]. [file gb-2009-10-5-r48-S8.doc]

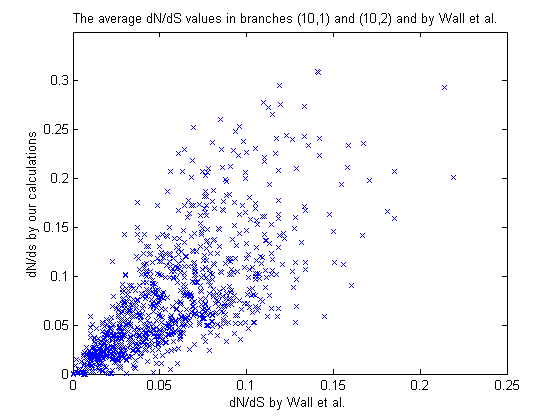


**Suppl. Fig. 3: ER values computed in our study vs. the ER values computed in Wall *et al*. The correlation between the two estimations is 0.75 (p-value < 10^-50^).**
